# Left Upper Lobectomy for Pulmonary Vein Occlusion After Catheter Ablation for Atrial Fibrillation

**DOI:** 10.1016/j.atssr.2024.10.023

**Published:** 2024-11-09

**Authors:** Nobuhisa Yamazaki, Yasuto Sakaguchi, Hirokazu Tanaka, Makoto Sonobe

**Affiliations:** 1 Department of Thoracic Surgery, Japanese Red Cross Osaka Hospital, Osaka, Japan

## Abstract

A 64-year-old man complained of recurrent bloody sputum 6 months after catheter ablation for atrial fibrillation. Contrast-enhanced chest computed tomography revealed left upper pulmonary vein occlusion. Due to challenges in revascularization, lung resection was performed. Although extensive adhesions were observed around the pulmonary hilum and pleura, a left upper lobectomy was successfully performed using a video thoracoscopic approach. The patient had no postoperative complaints. Pathologic findings showed irreversible lung damage, including lung fibrosis and intimal thickening of pulmonary blood vessels. These results indicate that resection is more appropriate than revascularization at the chronic phase of complete pulmonary vein occlusion.

Catheter ablation is a common and widespread treatment for symptomatic paroxysmal atrial fibrillation.^1^ Although it is a generally safe treatment, serious complications, including pulmonary vein stenosis or occlusion, cardiac tamponade, and left atrioesophageal fistula, can occur. Pulmonary vein stenosis or occlusion can cause severe hemoptysis and dyspnea, which may require therapeutic intervention. Catheter intervention, such as balloon dilation and stenting, is often performed for pulmonary vein stenosis or occlusion,^2^ whereas surgical treatment is rarely required. Herein, we report a patient who needed lung resection for pulmonary vein occlusion after catheter ablation for atrial fibrillation.

A 64-year-old man underwent catheter ablation for atrial fibrillation at another hospital. He visited our hospital 6 months after his ablation with complaints of left back pain and bloody sputum lasting 1 month. He was diagnosed as having pneumonia of the left upper lobe and pleuritis, and his symptoms improved after administration of antibiotics. Four months after his initial consultation, however, he came to our hospital complaining of intermittent bloody sputum. Contrast-enhanced computed tomography scans of the chest revealed an absent shadow for the left upper pulmonary vein, ground glass opacity, and consolidation at the left upper lobe (Figures 1A-1C). He was thus diagnosed with pulmonary vein occlusion, presumably a complication from previous catheter ablation. Surgical resection was indicated because of the difficulty in transcatheter revascularization for the pulmonary vein, with markedly decreased blood flow to the left upper lobe revealed by pulmonary perfusion scintigraphy (Figure 1D), and the presence of recurrent bloody sputum. Seven months after his initial consultation, a video-assisted thoracoscopic left upper lobectomy was performed. The intraoperative findings revealed extensive pleural adhesions, many enlarged capillaries in the pleura, and inflammatory changes at the pulmonary hilum. Dissection of the adhesions was difficult but possible. The upper pulmonary vein was cut using a surgical stapler outside the pericardium (Figure 2). Histopathologic findings revealed fibrous thickening of the pulmonary arteriovenous intima, collapse and fibrosis of the alveoli, and histological changes due to pulmonary hypertension and alveolar hemorrhage (Figure 3). The patient had an uneventful postoperative course, with no bloody sputum observed up to 2 months after surgery.

**Figure 1:**
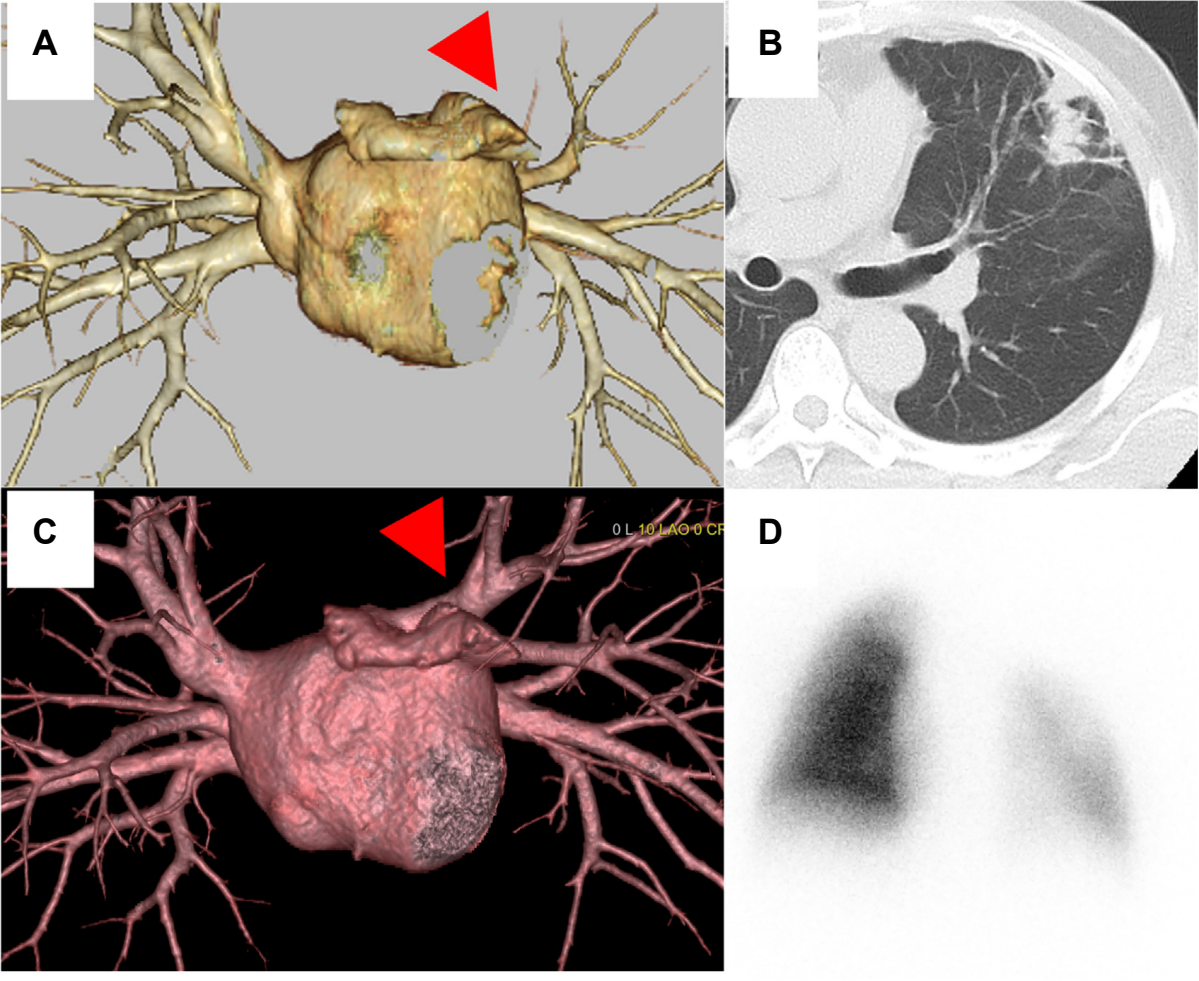
Preoperative image findings. Three-dimensional CT scans (A) before and (B) after catheter ablation. A shadow of the left upper pulmonary vein (red arrowheads) was absent. (C) Computed tomography findings in the lung revealed the consolidation with ground glass shadow in the left upper lung. (D) Pulmonary perfusion scintigraphy showed the reduced blood flow in the left upper lung.

**Figure 2:**
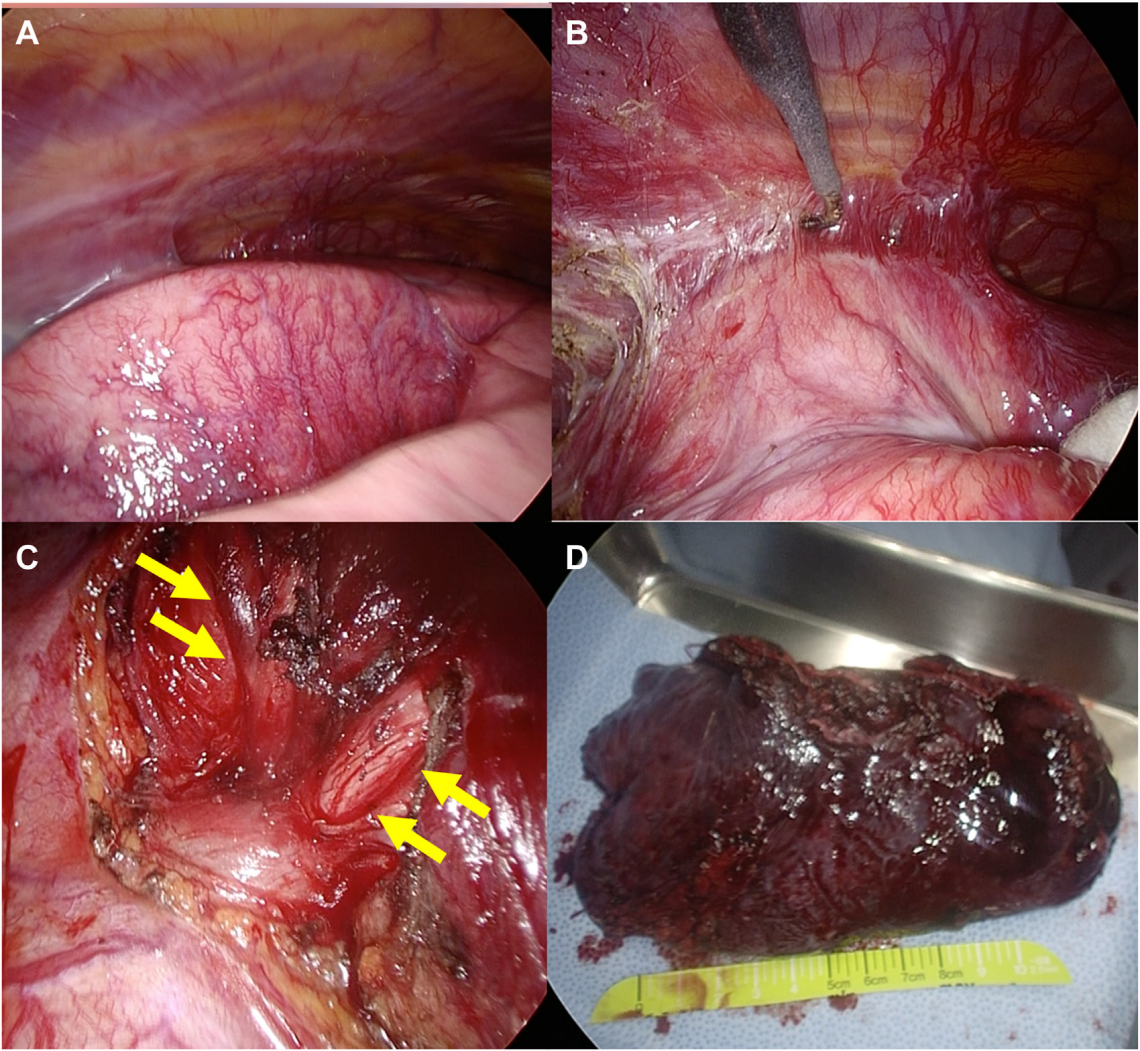
Intraoperative findings. The left upper lung showed the development of (A) neo-angiogenesis on the pleura and (B) the wide range of adhesion. (C) The tissue around the superior pulmonary vein (yellow arrows) exhibited inflammatory changes. (D) Resected left upper lobe.

**Figure 3:**
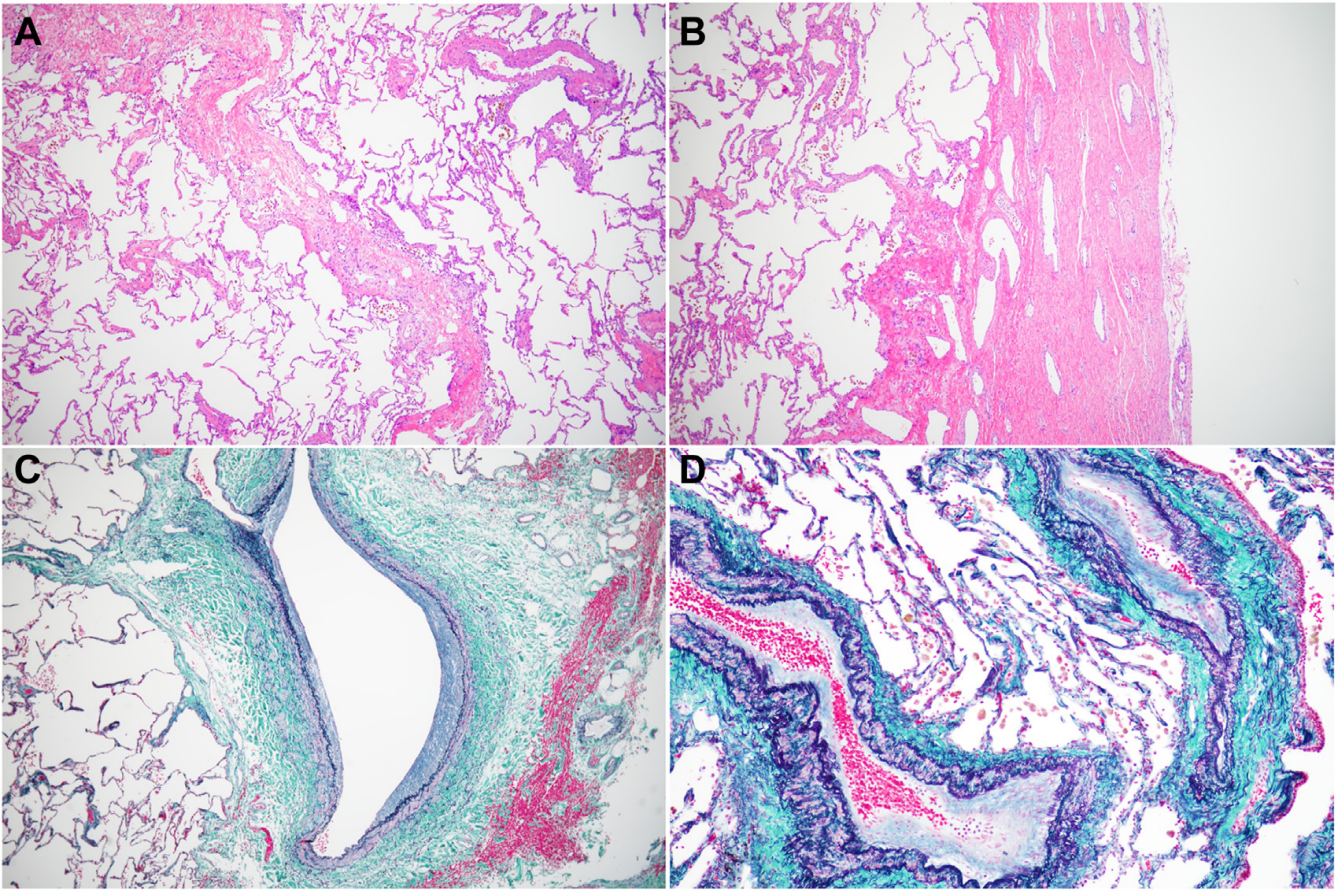
(A) Hematoxylin and eosin staining of the left lung (magnification ×40). Septal thickening, congestion, collapse, and fibrosis of the alveoli were observed. (B) Hematoxylin and eosin staining of the thickened visceral pleura (magnification ×40). Dilated vessels were formed in the pleura. Elastica-Masson staining of the pulmonary veins (C) and (D) arteries with intimal fibrous thickening (magnification ×40 and ×100, respectively).

## Comment

Catheter ablation is one of the main treatment modalities used for paroxysmal atrial fibrillation, along with drug therapy. Technological advances and the development of catheter devices have improved the treatment outcomes for paroxysmal atrial fibrillation,^1^ but serious complications, such as atrium-esophageal fistulae, cardiac tamponade, and pulmonary vein stenosis, can still occur.

The incidence of pulmonary vein stenosis or occlusion is still unclear because asymptomatic cases of pulmonary vein stenosis or occlusion can remain undetected. However, according to a worldwide survey from 85 centers, pulmonary vein stenosis or occlusion requiring intervention after catheter ablation for atrial fibrillation occurs in 0.29% of patients, and has been decreasing in recent years.^1^ The ablation procedure often involves the cauterization of the left atrium near the pulmonary vein orifice, which can result in pulmonary vein stenosis due to heat-induced collagen denaturation in the pulmonary vein itself.^3^ Scarring stenosis progresses slowly and causes severe stenosis or occlusion, which can lead to peripheral lung edema, fibrosis, infarction, and inflammatory reactions. This results in symptoms such as hemoptysis, dyspnea on exertion, cough, and fever, as demonstrated in our case study. Because of these symptoms, pulmonary vein stenosis or occlusion is often misdiagnosed as bacterial pneumonia or other common respiratory diseases.^2^ Thus, we should recognize the importance of confirming a history of catheter ablation for atrial fibrillation when we consider differential diagnoses for pulmonary vein stenosis or occlusion.

There is no consensus on the treatment of pulmonary vein stenosis or occlusion. Some patients are only followed up, while others are treated with catheter interventions such as balloon dilation or stenting. Catheter intervention is useful in that it avoids the need for highly invasive procedures, including lung resection or surgical revascularization. However, catheter intervention can potentially cause restenosis and lead to serious complications such as bleeding and cardiac tamponade.^2^ Surgical treatment may be indicated in cases where catheter intervention would be challenging due to severe or recurrent stenosis. Surgical treatment options include revascularization^4^ and lung resection. Although there has been a report of successful surgical revascularization, another report recommends the use of lung resection when there is irreversible damage to the pulmonary parenchyma.^5^ The lung resection procedure can often involve a left lung lobectomy due to central occlusion of the pulmonary veins and a higher frequency of ablation sites on the left side.^6^ In a previous report, intraoperative findings revealed marked congestive changes in the lung and inflammatory adhesions in the hilar region and pleura, while dilated capillaries had developed as collateral blood vessels on the visceral pleural surface.^7^ Pathologic results showed hemorrhage, infarction, and fibrosis in the lung, as well as intimal thickening of peripheral pulmonary vessels due to the fibrosis.^5^ These intraoperative and pathologic findings were observed in our patient as well.

In our case study, we opted for lung resection instead of revascularization due to complete occlusion of the upper pulmonary vein, which was revealed with pulmonary perfusion scintigraphy, and persistent complaints of recurrent hemoptysis. Our procedure was also instrumental in preventing the progression to lung deterioration. The revascularization procedure would not have been beneficial because of the severe damage to the pulmonary parenchyma and vessels that was revealed in pathologic findings in our case. Given the limited reports and the long-term postoperative outcomes for similar cases, the choice of appropriate treatment method remains controversial. However, lung resection may be a potentially effective treatment for patients in the chronic phase of severe pulmonary vein stenosis or occlusion.
